# *Mycoplasma genitalium* protein of adhesion inhibits human urethral epithelial cells apoptosis via CypA/PI3K/AKT/mTOR-dependent autophagy

**DOI:** 10.3389/fmicb.2025.1570659

**Published:** 2025-03-26

**Authors:** Li Chen, Dan Luo, Hua Xiao, Zhuo Zeng, Haodang Luo, Siqi Gao, Xiaoqian Tang, Zhijia Huang, Yanhua Zeng

**Affiliations:** ^1^Basic Medical School, Hengyang Medical College, Institute of Pathogenic Biology, University of South China, Hengyang, Hunan, China; ^2^Hunan Provincial Key Laboratory for Special Pathogens Prevention and Control, Hengyang, Hunan, China; ^3^Department of Clinical Laboratory, The Affiliated Nanhua Hospital, Hengyang Medical College, University of South China, Hengyang, Hunan, China; ^4^Department of Critical Care Medicine, The Second Affiliated Hospital, Hengyang Medical School, University of South China, Hengyang, Hunan, China

**Keywords:** *Mycoplasma genitalium*, MGPA, cyclophilin A, autophagy, apoptosis

## Abstract

**Background:**

*Mycoplasma genitalium*, a prokaryotic microorganism, is a known pathogen of sexually transmitted infections. Previously, we identified cyclophilin A (CypA) as the membrane receptor on human urethral epithelial cells (SV-HUC-1) that binds to the *M. genitalium* protein of adhesion (MgPa) and demonstrated that recombinant MgPa (rMgPa) inhibits apoptosis via CypA-mediated regulation of the PI3K/AKT/NF-κB pathway. Given the established interplay between autophagy and apoptosis, this study aims to investigate whether rMgPa inhibits apoptosis in SV-HUC-1 cells by modulating CypA/PI3K/AKT/mTOR-dependent autophagy.

**Methods:**

In this work, after SV-HUC-1 cells were stimulated with rMgPa, autophagy was detected using Western blotting, immunofluorescence and transmission electron microscopy, respectively. Western blotting and Annexin V/PI assays were used to determine the signaling pathway involved in rMgPa- inhibited apoptosis via inducing autophagy.

**Results:**

rMgPa upregulated the autophagy-related proteins ATG7 and LC3B while downregulating P62 expression in SV-HUC-1 cells. Transmission electron microscopy showed the presence of intracellular autophagosomes, and indirect immunofluorescence confirmed the enhanced expression of LC3B, indicating that rMgPa induces autophagy. Silencing of CypA significantly attenuated rMgPa-induced autophagy, highlighting the essential role of CypA in this process. Furthermore, rMgPa was found to regulate the PI3K/AKT/mTOR pathway via CypA, thereby promoting autophagy. Western blot analysis and Annexin V/PI assays confirmed that rMgPa-induced autophagy inhibits apoptosis in urothelial cells through a CypA-dependent mechanism.

**Conclusion:**

This study demonstrates that rMgPa suppresses apoptosis in SV-HUC-1 cells by inducing autophagy via CypA-mediated modulation of the PI3K/AKT/mTOR pathway, which elucidates a novel survival strategy employed by *M. genitalium* within host cells and provides valuable insights for potential therapeutic interventions targeting *M. genitalium* infections.

## 1 Introduction

*Mycoplasma genitalium*, a member of the class Mollicutes, is the smallest known self-replicating prokaryotic microorganism capable of growth in artificial media. *M. genitalium* was first isolated from the urethral discharges of two male patients who suffered from non-gonococcal urethritis (Tully et al., 1981) and the first microorganisms to be fully sequenced (Fookes et al., 2017). *M. genitalium* is implicated in sexually transmitted infections that, if left untreated, can lead to urethritis and prostatitis in males, infertility, pelvic inflammatory disease, and adverse pregnancy outcomes in females (Tamarelle et al., 2019; Jensen et al., 2022). Moreover, infection with *M. genitalium* has been associated with an increased susceptibility to human immunodeficiency virus (HIV) infection, highlighting its substantial public health impact (Das et al., 2014). Electron microscopy reveals that *M. genitalium* possesses a distinctive flask-shaped morphology with a specialized terminal tip structure rich in cytoadhesins and adhesion-associated proteins (Burgos et al., 2006). Genetic studies have demonstrated that both class I mutants (which express minimal levels of P140) and class II mutants (which lack P140) exhibit adhesion-negative phenotypes (Mernaugh et al., 1993). These findings establish the *M. genitalium* protein of adhesion (MgPa, also known as P140) as a critical factor for host cell adherence. Similar to other mycoplasmas such as *M. pneumoniae*, which can invade and replicate within human lung cancer cells leading to cellular vacuolation (Li et al., 2014), *M. genitalium* exhibits intracellular survival capabilities, persisting for up to 7 days within mammalian cells (Dallo and Baseman, 2000). Ueno et al. reported that *M. genitalium* can rapidly invade host cells, localizing to perinuclear regions and even entering the nucleus within 30 min post-infection confirming its classification as a facultative intracellular parasite (Ueno et al., 2008). Despite these insights, the mechanisms underlying *M. genitalium*’s intracellular survival remain incompletely understood.

Our previous research identified cyclophilin A (CypA) as the membrane receptor for MgPa on human urethral epithelial cells (SV-HUC-1), facilitating *M. genitalium* adhesion and invasion (Deng et al., 2018). CypA, a peptidyl-prolyl cis-trans isomerase, plays crucial roles in various cellular processes, including protein folding and immune regulation (Chen L. et al., 2024). It is also involved in the regulation of apoptosis, for instance, CypA stabilizes the Twist1 protein to regulate apoptosis in A549 cells (Wu et al., 2022) and modulates CD147-related pathways to inhibit apoptosis in MKN45 gastric cancer stem-like cells (Cho and Jung, 2023). We also confirmed that CypA is associated with recombinant MgPa (rMgPa) suppressing apoptosis in SV-HUC-1 cells (Liao et al., 2022).

Autophagy is an evolutionarily conserved catabolic process that maintains cellular and organismal homeostasis by degrading damaged organelles, invading pathogens, and protein aggregates via the lysosomal system (Bao et al., 2024; Chen D. et al., 2024). The balance between autophagy and apoptosis is critical for cell survival and is finely regulated under physiological conditions. Dysregulation of this balance is associated with various diseases, including acute brain injury (He et al., 2023) and Parkinson’s disease (Bekker et al., 2021). Studies substantiated that autophagy can inhibit apoptosis in certain contexts, such as during Largemouth Bass Ranavirus (LMBV) (Deng et al., 2022) and classical swine fever virus infections (Li et al., 2023), and myocardial ischemia–reperfusion injury (Han et al., 2024). Our previous study verified that rMgPa can regulate the PI3K/AKT/mTOR pathway via CypA to suppress SV-HUC-1 cell apoptosis (Liao et al., 2022). The PI3K/AKT/mTOR pathway is a central regulator of cell growth, proliferation, and survival, and it plays a pivotal role in autophagy regulation (Xu et al., 2020).

Given the intricate relationship between autophagy and apoptosis, we hypothesized that rMgPa might inhibit apoptosis in SV-HUC-1 cells by inducing autophagy through the PI3K/AKT/mTOR signaling pathway mediated by CypA. Therefore, in this study, we investigated the effect of rMgPa on autophagy and apoptosis in SV-HUC-1 cells and explored the underlying mechanisms involving CypA and the PI3K/AKT/mTOR pathway. Understanding how *M. genitalium* manipulates host cell survival mechanisms may provide valuable insights into its intracellular survival strategies and contribute to the development of novel therapeutic approaches targeting autophagy-related molecules for the treatment of *M. genitalium* infections.

## 2 Materials and methods

### 2.1 Preparation of recombinant MgPa protein

In our previous study, the immunodominant region of MgPa (comprising amino acid residues 1,075–1,364 of MgPa), which exhibits low homology to *M. pneumoniae* P1 protein and has strong immunogenicity, was selected and the PET-30a(+)/MgPa recombinant prokaryotic expression vector was successfully constructed. In this study, the rMgPa protein was expressed, purified, concentrated, and characterized according to the relevant conditions in our previous study (Deng et al., 2018), and then the protein was subsequently treated with polymyxin B to remove endotoxin before experimental use.

### 2.2 Cell culture

Human urethral epithelial cell (SV-HUC-1, ATCC TCHu169) was maintained at the Institute of Pathogenic Biology, University of South China. Cells were cultured using F-12 K medium (Gibco, United States) supplemented with fetal bovine serum (ExCell Bio, USA) at 37°C and 5% CO_2_.

### 2.3 Western blotting

Total cellular proteins were extracted using 100 μL cell lysis buffer (Kangwei Century, CW2333S, China) containing phosphatase and protease inhibitors (Sigma, United States). Protein concentration was determined using BCA protein assay (Thermo Fisher, United States) and then 30 μg/well of proteins were separated by SDS-PAGE and electrotransferred onto PVDF membranes. Antibodies used in this study are listed as follows: *β*-actin antibody (Cell Signaling Technology, 3700S), ATG7 antibody (Abcam, EPR6251), LC3B antibody(Cell Signaling Technology, 3,868 T), P62 antibody (Cell Signaling Technology, 8,025 T), PI3K Antibody (Jingjie Bio, PTM5198), phospho-PI3K Antibody (Bicentennial, AF5905), Akt Antibody (Jingjie Bio, PTM-6071), Phospho-Akt Antibody (Jingjie Bio, PTM-6649), mTOR (Proteintech, 66,888-1-Ig) and p-mTOR (Proteintech, 67,778-1-Ig), caspase-3 antibody (Immunoway, YT0656), cleaved-caspase-3 antibody (Immunoway, YM3431), anti-rabbit IgG-HRP antibody (Abcam, Ab270144) or anti-mouse IgG-HRP antibody (Cell Signaling Technology, 4408S). The PVDF membranes were first incubated with primary antibodies and then the corresponding HRP-conjuncted secondary antibodies, respectively. After were washed five times, the protein bands were visualized using the developer solution (Millipore, P36599, United States) and documented using the Western blotting system G: BOX Chemi XX9 (Syngene, United Kingdom). Band intensities were quantified using ImageJ software.

### 2.4 Transmission electron microscopy

Autophagic vesicles were examined in three experimental groups: untreated control, rMgPa-treated (20 μg/mL), and rapamycin-treated positive control (100 nmol/L, Solarbio). The culture medium in the bottle was discarded and 1 mL of electron microscopy fixative (Gluta fixative) was added immediately, and the cells were gently scraped with a cell scraper in the same direction without rinsing to avoid repeated scraping. The cells were collected and then centrifuged at 4, 000 *g* for 7 min. The fixative was then discard and replaced with new fixative to fix for 2 h at room temperature. The samples were transferred to 4°C for storage away from light and then transported to Changsha Xavier Biotechnology Co., Ltd. to carry out the transmission electron microscopy observation.

### 2.5 Immunofluorescence analysis

SV-HUC-1 cells exhibiting optimal growth conditions were taken and seeded in 24-well plates, and then treated with LC3B antibody (1:400) for 2 h at 37°C in a warm box. The plates were washed 3 times, and FITC-labeled sheep anti-mouse IgG antibody (Abcam, Ab102457,1:1,000) and DAPI (Beyotime, China) were added for nuclear staining, and then the plates were blocked with anti-fluorescence quencher. The fluorescence intensities of the above treated cells were observed using an inverted fluorescence microscope.

### 2.6 Transfection of small interfering RNA and mCherry-eGFP-LC3B autophagy double-label plasmid

CypA-siRNA (Origene, SR321383, United States) was purchased from Origene Technol-ogies, Inc. The sequence of CypA-siRNA is as follows: rUrGrUrUrUrUrUrCrArGrArArGrUrUrUrArCrCrCrUrUrU“rUrCrUrUrUr. The pCMV-mCherry-eGFP-LC3B autophagy reporter plasmid (Beyotime, China, D2816) was extracted using an endotoxin-free plasmid macro extraction kit (Kangwei Century, CW2108M) according to the manufacturer’s instructions. The target sequences were transfected into SV-HUC-1 cells using Lipofectamine 2000 in terms of the specification. Briefly, after waiting for the cells to grow to 50% ~ 60% confluence, the serum-free medium was used to renew the F-12 K Nutrient Mix, the CypA-siRNA double strand (20 nmol) and the pCMV-mCherry-eGFP-LC3B autophagy double-label plasmid (2 μg) duplex were, respectively, incubated for 20 min with 5 μL of Lipofectamine 2000 in serum-free medium at room temperature. The CypA-siRNA and plasmid duplex were then added to the cells for further incubation for 6–8 h, followed by the replacement with a complete medium. The cells were then incubated for 48 h for the next experiments. An inverted fluorescence microscope was then used for bright field imaging to observe the changes of autophagy flow.

### 2.7 Flow cytometric analysis of apoptosis

SV-HUC-1 cells were pre-transfected with or without CypA-siRNA or pretreated with or without 3-MA or STS, the cells were incubated with rMgPa for 24 h. Cells were then collected and dyed with FITC Annexin V and PI referring to the instructions of manufacturer (Abbkine, KTA0002, China). Analysis was performed using a BD Biosciences FACSCalibur™ flow cytometer, and data were processed using FlowJo software. Gating was set based on the following groups: the staurosporine group stained with PI (PI positive control), the staurosporine group stained with FITC Annexin V (Annexin V positive control), and the unstained group (negative control).

### 2.8 Statistical analysis

Results were expressed as mean ± standard deviation (SD) of three independent experiments. Statistical analyses were first executed using One-Way ANOVA and then the Student’s *t*-test was used between two groups, and all analyses were implemented using GraphPad Prism. *p* < 0.05 was considered statistically significant.

## 3 Results

### 3.1 rMgPa can promote SV-HUC-1 cell autophagy

**Figure 1:**
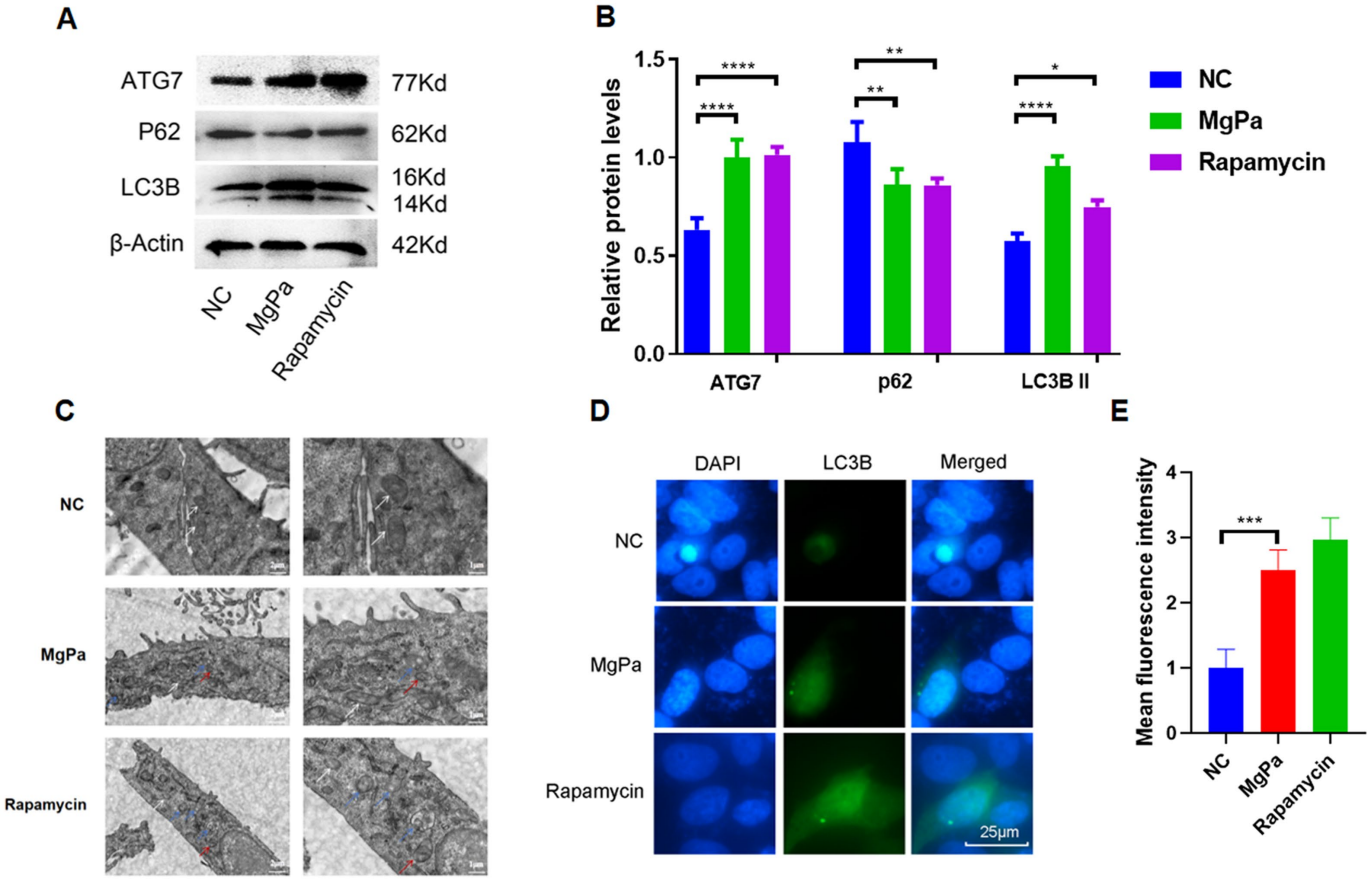
Detection of rMgPa-induced autophagy. **(A)** Western blot detection of the expressions of ATG7, LC3B, and P62. SV-HUC-1 cells were incubated with DMSO, rMgPa (10 and 20 μg/mL, respectively) or Rapamycin for 24 h, and the expressions of ATG7, LC3B and P62 were revealed in the representative WB gels and summarized data. **(B)** The relative protein levels of the Western blot band. The gray values of ATG7, LC3B, and P62 were counted by GraphPad Prism 8. ^*^p < 0.05, ^**^p < 0.01, ^***^p < 0.001, ^****^p < 0.0001 for significant differences between two compared groups. **(C)** Detection of rMgPa-induced autophagy by transmission electron microscopy. SV-HUC-1 cells were cultured with rMgPa or Rapamycin for 24 h, and autophagosomes and autophagolysosomes were observed under transmission electron microscope (5,000× on the left and 10,000× on the right). Different arrow colors were used in the figure to indicate intracellular components, white for mitochondria, red for autophagosomes, and blue for autophagolysosomes. Bar, 1 μm. **(D)** Indirect immunofluorescence analysis of LC3B expression. SV-HUC-1 cells were incubated with rMgPa or rapamycin for 24 h, and the fluorescence was observed under an inverted fluorescence microscope (green is LC3B-specific fluorescence, Bar, 25 μm). **(E)** The mean fluorescence intensity of LC3B was counted with GraphPad Prism 8. ^*^p < 0.05, ^**^p < 0.01, ^***^p < 0.001 for significant differences between two compared groups.

To explore the influence of rMgPa on autophagy in SV-HUC-1 cells, Western blot was implemented to examine the expressions of autophagy-related molecules ATG7, LC3B, and P62. Figures 1A,B demonstrated that the expressions of ATG7 and LC3B in rMgPa-treated cells were remarkably higher than that of the control cell, whereas the expression of P62 was notably diminished in the rMgPa-treated cells (*P <* 0.01). And the changing trends of ATG7, LC3B, and P62 in rMgPa-treated cells were consistent with those of the Rapamycin-treated group. Transmission electron microscopy provided further evidence of autophagy induction. As depicted in Figure 1C, rMgPa-treated SV-HUC-1 cells exhibited prominent autophagic vesicle formation and an increased number of autophagolysosomes, similar to the rapamycin-treated group. In contrast, the control group displayed no autophagic vesicles or autophagolysosomes, showing only normal organelles such as mitochondria. Indirect immunofluorescence analysis corroborated these findings. Figure 1D illustrates that the fluorescence intensity of LC3B was significantly enhanced in rMgPa-treated cells compared to control cells (*p* < 0.001), indicating increased autophagic activity. Collectively, these results demonstrate that rMgPa promotes autophagy in SV-HUC-1 cells.

### 3.2 rMgPa promotes SV-HUC-1 cell autophagy via CypA

**Figure 2:**
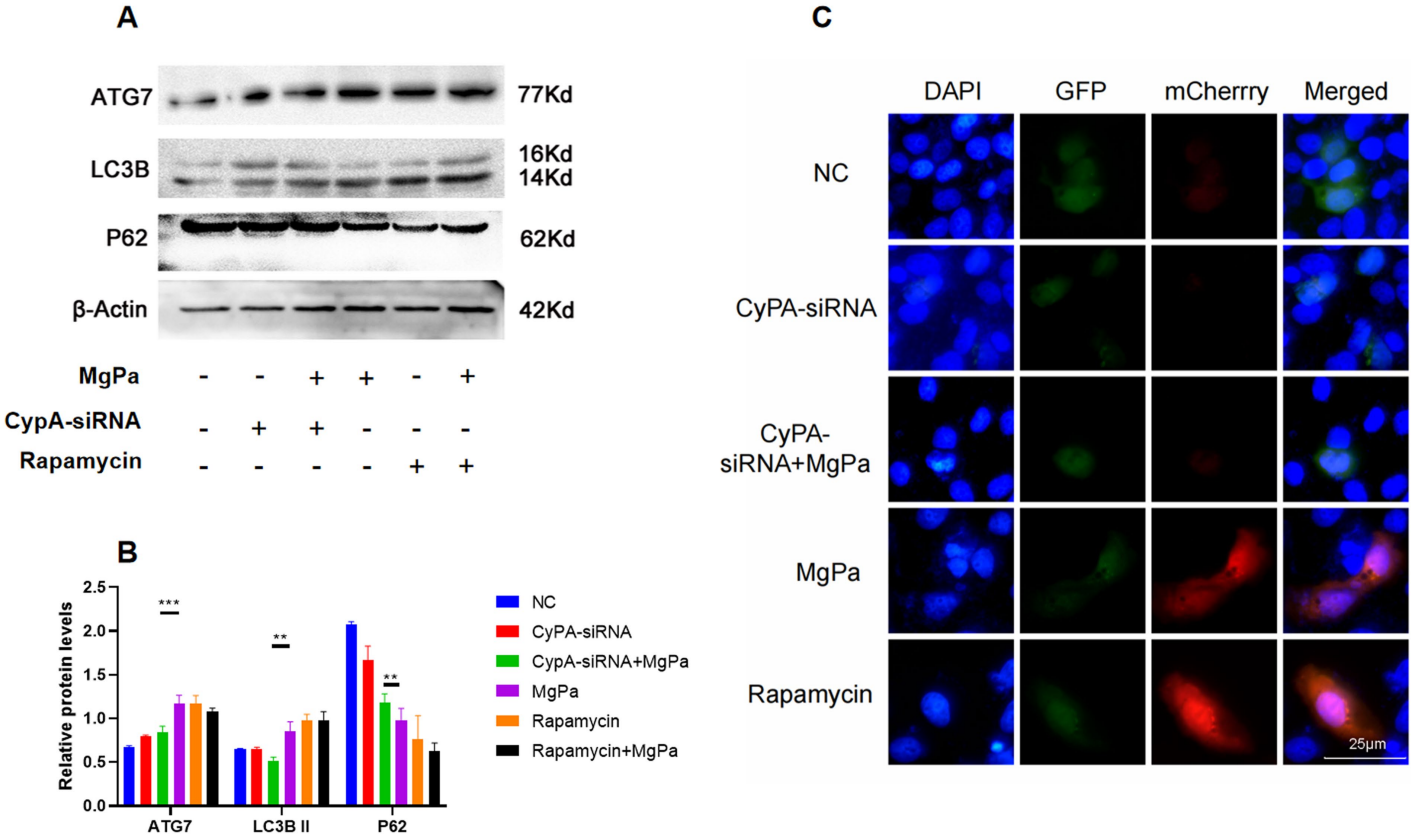
Detection on the effect of CypA on rMgPa-induced autophagy. **(A)** Western blot detection of proteins expression. SV-HUC-1 cells were incubated with rMgPa or rapamycin for 24 h after preincubation with or without CypA-siRNA, and representative WB gels and pooled data showed the expression levels of ATG7, LC3B, and P62. **(B)** The relative protein levels of the band of Western blot. The grayscale values of ATG7, LC3B II and P62 were counted using GraphPad Prism 8. ^*^p < 0.05, ^**^p < 0.01, ^***^p < 0.001 for significant differences between two compared groups. **(C)** Immunofluorescence analysis on the effect of CypA on rMgPa-induced autophagic flow. SV-HUC-1 cells were transfected with mCherry-GFP-LC3B plasmid and CypA-siRNA, respectively, then incubated with rMgPa or rapamycin for 24 h, and fluorescence phenomenon (autophagic lysosomes in red, autophagic vesicles in green) was observed under an inverted fluorescence microscope. Bar: 25 μm.

To determine whether rMgPa induces autophagy through cyclophilin A (CypA), we examined the expression of ATG7, LC3B, and P62 proteins before and after CypA-siRNA transfection using Western blot analysis. As shown in Figures 2A,B, knockdown of CypA significantly attenuated the rMgPa-induced increase in ATG7 and LC3B expression (*p* < 0.01) and reversed the decrease in P62 expression (*p* < 0.05). Immunofluorescence analysis further supported these results. Figure 2C shows that the fluorescence intensity of autophagic lysosomes was markedly reduced in rMgPa-treated cells transfected with CypA-siRNA compared to cells treated with rMgPa alone. Additionally, rMgPa-treated cells exhibited significantly enhanced autophagic lysosome fluorescence compared to control cells, similar to the rapamycin-treated group. These findings suggest that rMgPa promotes autophagy in SV-HUC-1 cells via a CypA-dependent mechanism.

### 3.3 rMgPa can regulate the PI3K/AKT/mTOR pathway

**Figure 3:**
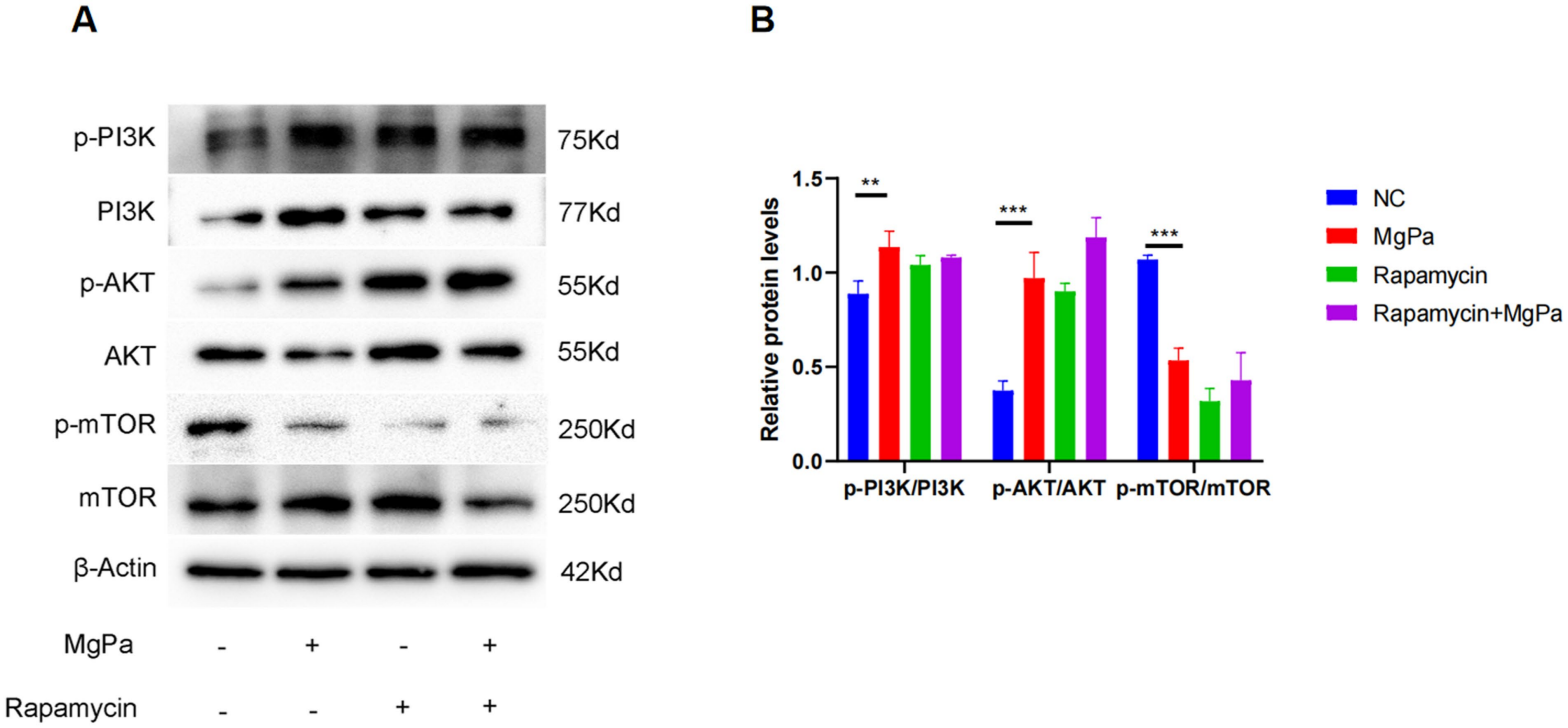
Western blot detection of PI3K/AKT and mTOR expressions and their phosphorylation. **(A)** Western blot detection. Protein expressions of p-PI3K/PI3K, p-AKT/AKT, p-mTOR/mTOR were detected in SV-HUC-1 cells stimulated for 24 h with rMgPa, rMgPa and Rapamycin, or Rapamycin, respectively. **(B)** The relative protein levels of the Western blot band. The gray values of p-PI3K/PI3K, p-AKT/AKT, p-mTOR/mTOR were counted with GraphPad Prism 8. ^*^p < 0.05, ^**^p < 0.01, ^***^p < 0.001 for significant differences between two compared groups.

We next investigated whether rMgPa influences the PI3K/AKT/mTOR signaling pathway to induce autophagy. Western blot analysis was used to assess the expression and phosphorylation levels of key proteins in this pathway. As shown in Figures 3A,B, rMgPa-treated cells exhibited significantly increased phosphorylation of PI3K (*p* < 0.01) and AKT (*p* < 0.001) compared to control cells. Conversely, the phosphorylation level of mTOR was significantly decreased in rMgPa-treated cells (*p* < 0.001). These results indicate that rMgPa activates the PI3K/AKT pathway while inhibiting mTOR phosphorylation.

### 3.4 rMgPa regulates the PI3K/AKT/mTOR pathway via CypA

**Figure 4:**
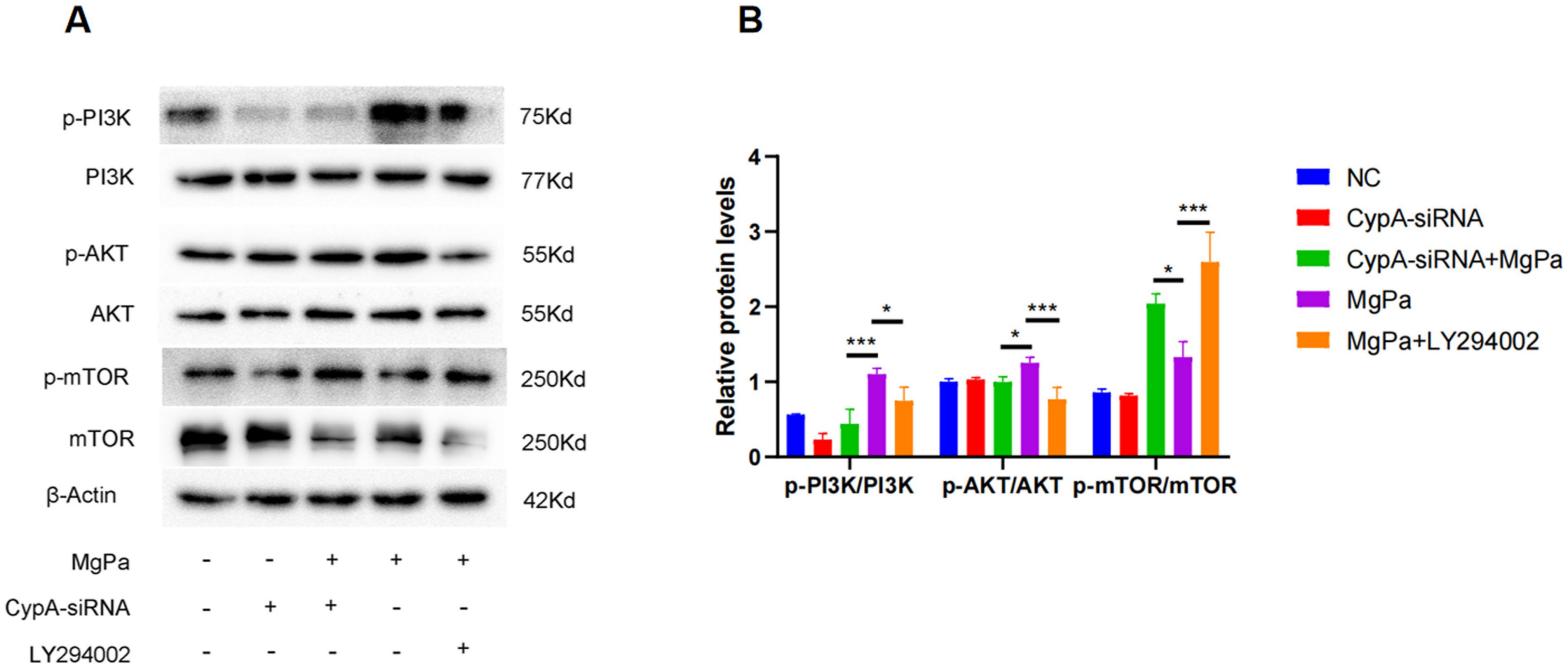
Detection of the effect of CypA on PI3K/AKT and mTOR expressions and responding phosphorylation. **(A)** Western blot detection. Protein expressions of p-PI3K/PI3K, p-AKT/AKT, and p-mTOR/mTOR in rMgPa-treated SV-HUC-1 cells transfected with CypA-siRNA or pretreated with PI3K inhibitor LY294002. SV-HUC-1 cells were incubated with rMgPa after pretreatment with or without the PI3K inhibitor LY294002. **(B)** The relative protein levels of the Western blot band. The gray values of p-PI3K/PI3K, p-AKT/AKT, and p-mTOR/mTOR were counted with GraphPad Prism 8. ^*^p < 0.05, ^**^p < 0.01, ^***^p < 0.001 for significant differences between two compared groups.

To ascertain whether the modulation of the PI3K/AKT/mTOR pathway by rMgPa is mediated through CypA, SV-HUC-1 cells were pretreated with the PI3K inhibitor LY294002 or transfected with CypA-siRNA before rMgPa stimulation. As presented in Figures 4A,B, both LY294002 treatment and CypA knockdown significantly reduced the rMgPa-induced phosphorylation of PI3K and AKT. In contrast, the phosphorylation level of mTOR was significantly increased compared to cells treated with rMgPa alone. These findings demonstrate that rMgPa regulates the PI3K/AKT/mTOR pathway via CypA.

### 3.5 rMgPa regulates PI3K/AKT/mTOR via CypA to promote autophagy in SV-HUC-1 cells

**Figure 5:**
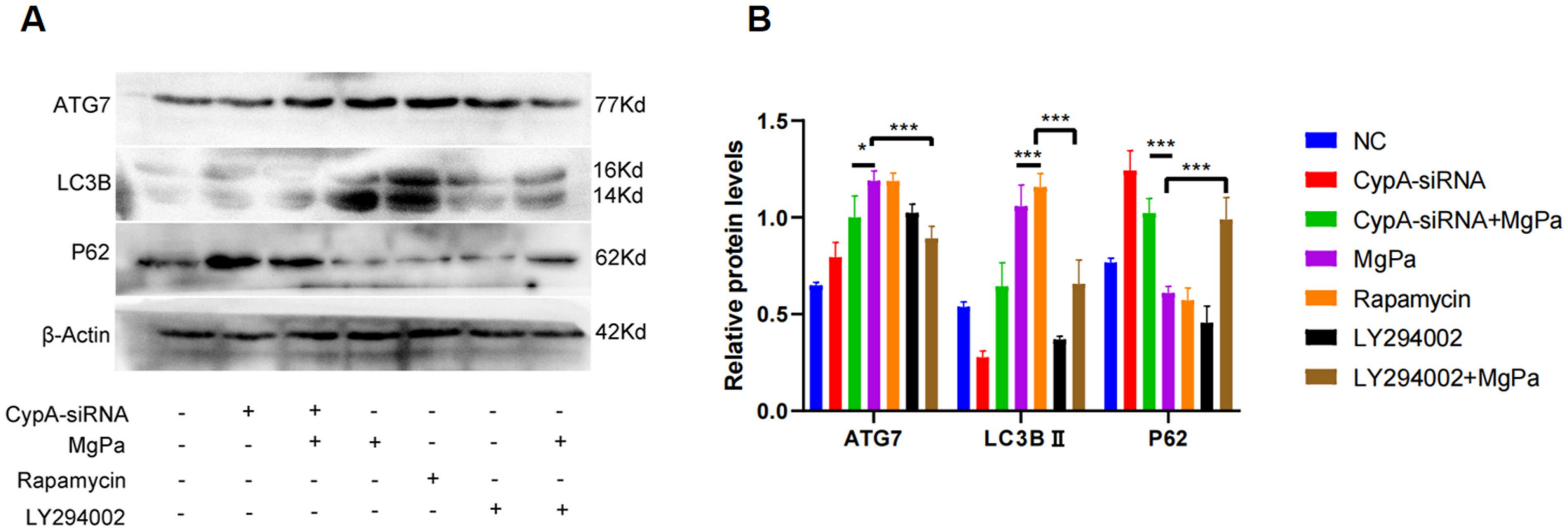
Detection of the effects of CypA-siRNA and LY294002 on the expressions of ATG7, LC3B and P62. **(A)** Western blot detection of ATG7, LC3B, and P62 expressions. SV-HUC-1 cells were pre-transfected with or without CypA-siRNA or pretreated with or without LY294002 and then incubated with rMgPa or rapamycin for 24 h, respectively. Representative WB gels and pooled data showed the ATG7, LC3B, and P62 protein expression levels. **(B)** The relative protein levels of the band of ATG7, LC3B and P62. The grayscale values of ATG7, LC3B, and P62 were counted using GraphPad Prism 8. ^*^p < 0.05, ^**^p < 0.01, ^***^p < 0.001 for significant differences between two compared groups.

To further elucidate the mechanism by which rMgPa induces autophagy, we assessed the expression of autophagy-related proteins after pretreatment with LY294002 or CypA-siRNA followed by rMgPa stimulation. As shown in Figures 5A,B, rMgPa-treated cells exhibited significantly higher expression levels of ATG7 and LC3B-II and a concomitant reduction in P62 expression compared to cells pretreated with LY294002 or transfected with CypA-siRNA (*p* < 0.001). These results confirm that rMgPa promotes autophagy in SV-HUC-1 cells by modulating the PI3K/AKT/mTOR pathway via CypA.

### 3.6 rMgPa promotes autophagy and thus inhibits SV-HUC-1 cell apoptosis via CypA

**Figure 6:**
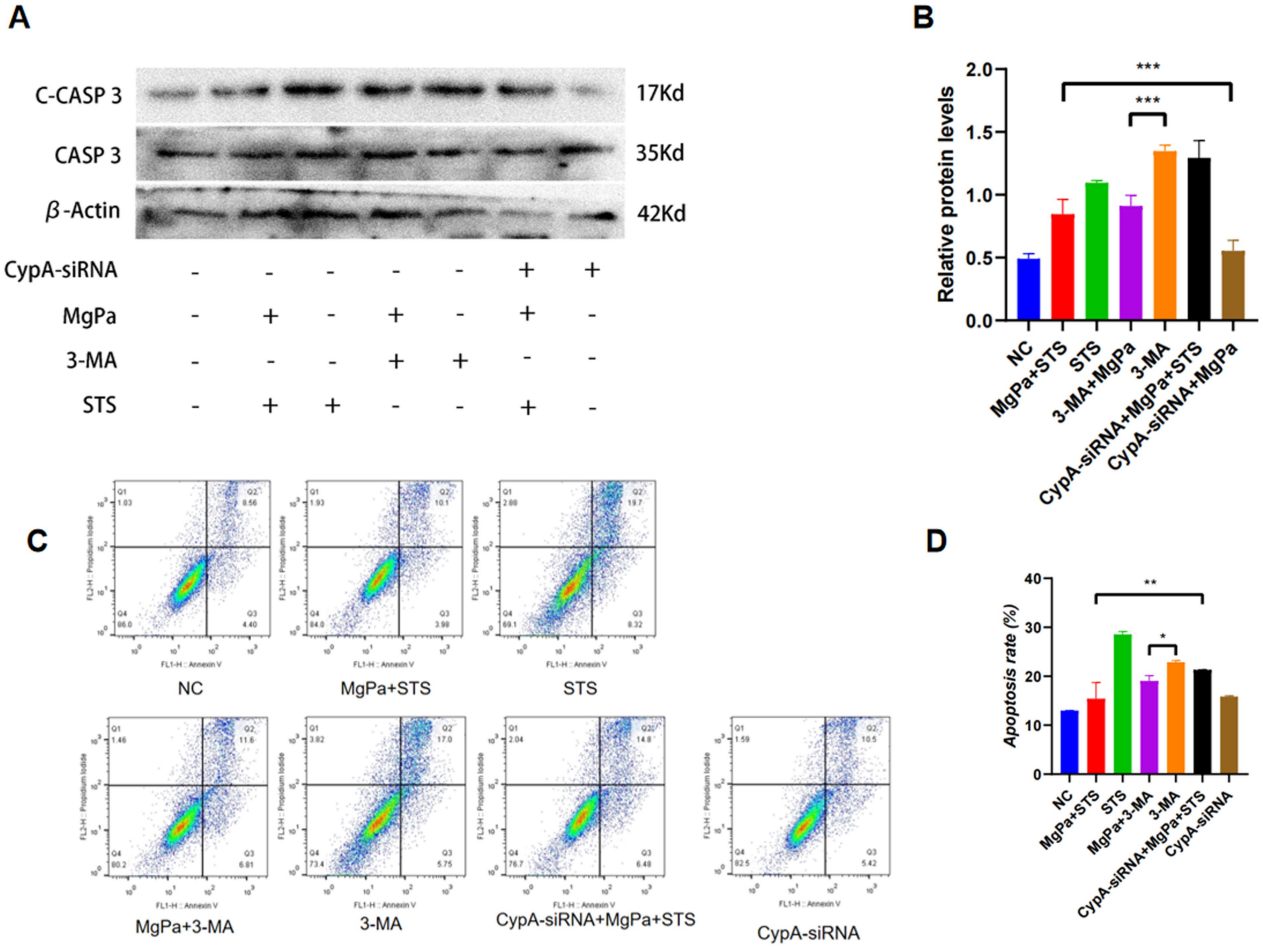
Detection of rMgPa inhibited-apoptosis by inducing autophagy. **(A)** Western blot detection of caspase-3 and cleaved Caspase-3 expressions. SV-HUC-1 cells were pretransfected with or without CypA-siRNA or pretreated with or without 3-MA or STS, the cells were incubated with rMgPa for 24 h, and representative WB gels and pooled data demonstrated the protein expression levels of Cleaved Caspase-3 and Caspase-3. **(B)** The relative protein levels of caspase-3 and cleaved Caspase-3. Grayscale values of Cleaved Caspase-3 were counted using GraphPad Prism 8. ^*^p < 0.05, ^**^p < 0.01, ^***^p < 0.001 for significant differences between two compared groups. **(C)** Flow cytometry detection. A bank control group and different treatment groups were set up, and the inhibitory effect of rMgPa on the apoptosis by promoting cellular autophagy was detected by flow cytometry. **(D)** The apoptosis rate was calculated according to the relevant experiments. ^*^p < 0.05, ^**^p < 0.01 for significant differences between two compared groups.

To investigate whether rMgPa inhibits apoptosis by inducing autophagy, we analyzed the expression of apoptosis-related proteins caspase-3 and cleaved caspase-3 after pretreating cells with the autophagy inhibitor 3-MA. Figures 6A,B show that 3-MA-treated cells exhibited a significantly higher expression of cleaved caspase-3 compared to the negative control, indicating increased apoptosis. However, cells co-treated with 3-MA and rMgPa displayed a significant reduction in cleaved caspase-3 expression compared to cells treated with 3-MA alone (*p* < 0.001), suggesting that rMgPa inhibits apoptosis by promoting autophagy. Furthermore, in cells transfected with CypA-siRNA and treated with rMgPa and staurosporine (STS), the expression of cleaved caspase-3 was significantly higher than in cells treated with rMgPa and STS (*p* < 0.001). Flow cytometry analysis corroborated these findings. As depicted in Figures 6C,D, the apoptosis rate in 3-MA-treated cells was 22.75%, significantly higher than in cells co-treated with 3-MA and rMgPa (18.41%). Additionally, cells transfected with CypA-siRNA and treated with rMgPa and STS exhibited a higher apoptosis rate (21.28%) compared to cells treated with rMgPa and STS (12.96%) (*p* < 0.001). These results collectively demonstrate that rMgPa inhibits apoptosis in SV-HUC-1 cells by promoting autophagy through CypA-mediated regulation of the PI3K/AKT/mTOR pathway.

## 4 Discussion

Since its first isolation in 1980, *M. genitalium* has emerged as a significant pathogen responsible for acute and chronic non-gonococcal urethritis and other reproductive tract infections transmitted sexually contact. Its capacity to co-infect with other sexually transmitted pathogens—including *Trichomonas vaginalis*, *Neisseria gonorrhoeae*, and *Chlamydia trachomatis*—augments host susceptibility to these infections (Kirkoyun Uysal et al., 2023). Notably, studies involving high-risk populations in the United States have reported co-infection rates of 29.9% with *C. trachomatis* and 23.6% with *N. gonorrhoeae* (Trent et al., 2018), underscoring the importance of including *M. genitalium* in sexually transmitted infection diagnostics. Moreover, emerging evidences have associated *M. genitalium* infection with ovarian cancer and high-risk human papillomavirus infections (Fortner et al., 2019), highlighting its potential role in oncogenesis. Despite these findings, the comprehensive understanding of *M. genitalium*’s infection course and its broader impact on host health remains incomplete. The pathogenicity of *M. genitalium* is partly attributed to its ability to adhere to, invade, and replicate within host cells, classifying it as a facultative intracellular parasite (Dallo and Baseman, 2000; Ueno et al., 2008). This intracellular lifestyle allows *M. genitalium* to evade the clearance of the host immune system and establish persistent infections. Understanding the mechanisms underlying its intracellular survival is crucial for elucidating its pathogenic strategies and developing targeted therapeutic interventions.

Cyclophilin A (CypA), a peptidyl-prolyl isomerase found on the membranes of both prokaryotic and eukaryotic cells, plays multifaceted roles in various cellular processes. Beyond its classical function in immunosuppression through cyclosporin A binding and thus inhibiting T-cell activation (Peng et al., 2024). CypA is also involved in protein folding, immune regulation, and modulation of inflammatory responses (Cho and Jung, 2023; Kalinina et al., 2023). Notably, CypA exhibits context-dependent effects on cell survival: it can inhibit apoptosis via regulation of the PI3K/AKT/mTOR signaling pathway (Ma et al., 2021), while also potentially promoting apoptosis through interactions with apoptosis-inducing factors (Wang X. et al., 2022). Therefore, the regulation of CypA on the cell cycle at different times changes accordingly. Our previous studies identified CypA as the membrane receptor for the adhesin MgPa on SV-HUC-1 cells (Deng et al., 2018) and demonstrated that recombinant MgPa (rMgPa) inhibits uroepithelial cell apoptosis through CypA-mediated activation of the PI3K/AKT/NF-κB pathway (Liao et al., 2022).

Autophagy is a conserved cellular process that maintains homeostasis through the degradation and recycling of cytoplasmic components. It is known that moderate cellular autophagy can maintain the balance of cellular survival, while excessive autophagy will lead to cell death, which demonstrates autophagy and apoptosis are naturally closely related (Miller and Thorburn, 2021). The delicate balance between autophagy and apoptosis is critical, as these processes are interconnected (Khoo et al., 2023; Wang et al., 2024). For instance, it has been reported that excessive apoptosis and senescence occurred when the level of autophagy in chondrocytes was reduced, and vice versa, the senescence and apoptosis of chondrocytes were inhibited when autophagy was increased after overexpression of the transcription factor EB (Liu et al., 2023). *Largemouth bass ranavirus* induces autophagy in carp epithelial tumor cells, whereas further enhancement of autophagy inhibits cell apoptosis (Deng et al., 2022; Li et al., 2023). Our previous study confirmed that rMgPa inhibits apoptosis in urothelial cells via the CypA-modulated PI3K/AKT/NF-κB pathway (Liao et al., 2022). Therefore, this study aims to investigate whether rMgPa induces autophagy via CypA and thus restrains SV-HUC-1 cell apoptosis to favor the intracellular survival of *M. genitalium* in the early stage of infection.

When a pathogen invades a host cell, the cell may initiate a series of responses as a result, including the activation of some signal pathways and the occurrence of corresponding changes. As for apoptosis, when the body is invaded by a pathogen, the body will generate both pro-apoptotic and anti-apoptotic signals, and the balance of these two signals can be easily broken, and the conditioning of the stimulating signals and the magnitude of the impact will determine whether the cell is heading toward apoptosis. Intracellular pathogens have evolved strategies to exploit host autophagy pathways for their benefit. For example, tick-borne bacteria can secrete effector proteins interacting with Beclin-1 to recruit autophagosomes into vesicles, facilitating their survival and replication within host cells (Wang and Cull, 2022). *Ehrlichia chaffeeusis*, the causative agent of human ehrlichiosis, is also able to induce cellular autophagy, thereby transferring autophagosomes into vesicles to acquire nutrients (Lin et al., 2016), which is the manner of maintaining their survival for most intracellular parasitic pathogens. In addition, such pathogens can keep the integrality and sustainability of their replication cycle by inhibiting apoptosis within host cells. In the present study, we demonstrated that rMgPa induces autophagy in SV-HUC-1 cells, as evidenced by increased expressions of autophagy-related molecules and formation of autophagosomes and autophagolysosomes.

Phosphoinositide 3-kinase (PI3K), serine/threonine protein kinase B (AKT), and mammalian target of rapamycin (mTOR) are vital signaling molecules responsible for the intracellular regulation of cell growth, metabolism, proliferation, and survival. Activation of PI3K leads to phosphorylation of AKT, which in turn can inhibit mTOR activity (Wang J. et al., 2022). In contrast, mTOR consists of mTOR complex 1 (mTORC1) and mTORC2, in which the mTORC1 negatively regulates autophagy by inhibiting the ULK1 complex. Studies proved that the PI3K/AKT/mTOR pathway modulates autophagy through key molecules such as Beclin-1, P62, and phosphatidylinositol 3-kinase class 3 (VPS34) (Ersahin et al., 2015). Therefore, this study focused on whether rMgPa can modulate the PI3K/AKT/mTOR pathway via CypA to promote autophagy in SV-HUC-1 cells. Our results indicate that rMgPa activates the PI3K/AKT pathway while inhibiting mTOR phosphorylation, thus promoting autophagy. The use of the PI3K inhibitor LY294002 attenuated these effects, and silencing of CypA expression negatively impacted the activation of the PI3K/AKT/mTOR pathway by rMgPa. These findings confirm that CypA mediates the modulation of this pathway by rMgPa.

Importantly, we found that rMgPa-induced autophagy contributes to the inhibition of apoptosis in SV-HUC-1 cells. The interplay between autophagy and apoptosis is complex, however, our data suggest that rMgPa promotes SV-HUC-1 cells autophagy via the CypA-mediated PI3K/AKT/mTOR pathway. This mechanism likely facilitates the intracellular survival and persistence of *M. genitalium* during the early stages of infection. In conclusion, our study unveils a novel mechanism by which *M. genitalium* promotes its intracellular survival. The adhesin MgPa interacts with host cell CypA to activate the PI3K/AKT/mTOR signaling pathway, leading to increased autophagy and reduced apoptosis in uroepithelial cells. These insights enhance our understanding of *M. genitalium* pathogenesis and may inform the development of targeted therapies aimed at disrupting these interactions to combat infections more effectively. However, the focus of this study has been on establishing the fundamental mechanisms and initial evidence through *in vitro* cell-based experiments. While these results are promising, we recognize that extending our findings to *in vivo* settings using animal model is a critical next step.

## 5 Conclusion

The results of this study demonstrate that rMgPa can suppress SV-HUC-1 cell apoptosis by modulating PI3K/Akt/mTOR pathway-induced autophagy via CypA.

## Data Availability

The original contributions presented in the study are included in the article/Supplementary material, further inquiries can be directed to the corresponding authors.

## References

[ref1] Bao Z. Wang P. Li Y. Ding H. Wen J. Zou K.. (2024). EphrinB2-mediated chondrocyte autophagy induces post-traumatic arthritis via rupture of cartilage homeostasis. J. Cell. Mol. Med. 28:e70095. doi: 10.1111/jcmm.70095, PMID: 39289794 PMC11408268

[ref2] Bekker M. Abrahams S. Loos B. Bardien S. (2021). Can the interplay between autophagy and apoptosis be targeted as a novel therapy for Parkinson’s disease? Neurobiol. Aging 100, 91–105. doi: 10.1016/j.neurobiolaging.2020.12.013, PMID: 33516928

[ref3] Burgos R. Pich O. Q. Ferrer-Navarro M. Baseman J. B. Querol E. Piñol J. (2006). *Mycoplasma genitalium* P140 and P110 cytadhesins are reciprocally stabilized and required for cell adhesion and terminal-organelle development. J. Bacteriol. 188, 8627–8637. doi: 10.1128/jb.00978-06, PMID: 17028283 PMC1698224

[ref4] Chen D. Wu L. Liu X. Wang Q. Gui S. Bao L.. (2024). *Helicobacter pylori* CagA mediated mitophagy to attenuate the NLRP3 inflammasome activation and enhance the survival of infected cells. Sci. Rep. 14:21648. doi: 10.1038/s41598-024-72534-5, PMID: 39289452 PMC11408507

[ref5] Chen L. Zeng Z. Luo H. Xiao H. Zeng Y. (2024). The effects of CypA on apoptosis: potential target for the treatment of diseases. Appl. Microbiol. Biotechnol. 108:28. doi: 10.1007/s00253-023-12860-2, PMID: 38159118 PMC13496919

[ref6] Cho H. J. Jung H. J. (2023). Cyclophilin a inhibitors suppress proliferation and induce apoptosis of MKN45 gastric Cancer stem-like cells by regulating CypA/CD147-mediated signaling pathway. Int. J. Mol. Sci. 24:4734. doi: 10.3390/ijms24054734, PMID: 36902161 PMC10003193

[ref7] Dallo S. F. Baseman J. B. (2000). Intracellular DNA replication and long-term survival of pathogenic mycoplasmas. Microb. Pathog. 29, 301–309. doi: 10.1006/mpat.2000.0395 11031124

[ref8] Das K. De la Garza G. Siwak E. B. Scofield V. L. Dhandayuthapani S. (2014). *Mycoplasma genitalium* promotes epithelial crossing and peripheral blood mononuclear cell infection by HIV-1. Int. J. Infect. Dis. 23, 31–38. doi: 10.1016/j.ijid.2013.11.022, PMID: 24661929 PMC4979978

[ref9] Deng X. Dai P. Yu M. Chen L. Zhu C. You X.. (2018). Cyclophilin a is the potential receptor of the *Mycoplasma genitalium* adhesion protein. Int. J. Med. Microbiol. 308, 405–412. doi: 10.1016/j.ijmm.2018.03.001, PMID: 29551599

[ref10] Deng L. Feng Y. OuYang P. Chen D. Huang X. Guo H.. (2022). Autophagy induced by largemouth bass virus inhibits virus replication and apoptosis in epithelioma papulosum cyprini cells. Fish Shellfish Immunol. 123, 489–495. doi: 10.1016/j.fsi.2022.03.026, PMID: 35364259

[ref11] Ersahin T. Tuncbag N. Cetin-Atalay R. (2015). The PI3K/AKT/mTOR interactive pathway. Mol. BioSyst. 11, 1946–1954. doi: 10.1039/c5mb00101c, PMID: 25924008

[ref12] Fookes M. C. Hadfield J. Harris S. Parmar S. Unemo M. Jensen J. S.. (2017). *Mycoplasma genitalium*: whole genome sequence analysis, recombination and population structure. BMC Genomics 18:993. doi: 10.1186/s12864-017-4399-6, PMID: 29281972 PMC5745988

[ref13] Fortner R. T. Terry K. L. Bender N. Brenner N. Hufnagel K. Butt J.. (2019). Sexually transmitted infections and risk of epithelial ovarian cancer: results from the Nurses’ health studies. Br. J. Cancer 120, 855–860. doi: 10.1038/s41416-019-0422-9, PMID: 30894687 PMC6474309

[ref14] Han X. Jiang Z. Hou Y. Zhou X. Hu B. (2024). Myocardial ischemia-reperfusion injury upregulates nucleostemin expression via HIF-1α and c-Jun pathways and alleviates apoptosis by promoting autophagy. Cell Death Discov 10:461. doi: 10.1038/s41420-024-02221-x, PMID: 39477962 PMC11525682

[ref15] He C. Xu Y. Sun J. Li L. Zhang J. H. Wang Y. (2023). Autophagy and apoptosis in acute brain injuries: from mechanism to treatment. Antioxid. Redox Signal. 38, 234–257. doi: 10.1089/ars.2021.0094, PMID: 35579958

[ref16] Jensen J. S. Cusini M. Gomberg M. Moi H. Wilson J. Unemo M. (2022). 2021 European guideline on the management of *Mycoplasma genitalium* infections. J. Eur. Acad. Dermatol. Venereol. 36, 641–650. doi: 10.1111/jdv.17972, PMID: 35182080

[ref17] Kalinina A. Tilova L. Kirsanov K. Lesovaya E. Zhidkova E. Fetisov T.. (2023). Secreted cyclophilin a is non-genotoxic but acts as a tumor promoter. Toxicology 500:153675. doi: 10.1016/j.tox.2023.153675, PMID: 37993081

[ref18] Khoo S. H. Wu P. R. Yeh K. T. Hsu S. L. Wu C. H. (2023). Biological and clinical significance of the AGE-RAGE axis in the aggressiveness and prognosis of prostate cancer. J. Food Drug Anal. 31, 664–682. doi: 10.38212/2224-6614.3475, PMID: 38526823 PMC10962675

[ref19] Kirkoyun Uysal H. Koksal M. O. Sarsar K. Ilktac M. Isik Z. Akgun Karapinar D. B.. (2023). Prevalence of *Chlamydia trachomatis*, Neisseria gonorrhoeae, and *Mycoplasma genitalium* among patients with urogenital symptoms in Istanbul. Healthcare 11:930. doi: 10.3390/healthcare11070930, PMID: 37046856 PMC10094226

[ref20] Li S. Li X. Wang Y. Yang J. Chen Z. Shan S. (2014). Global secretome characterization of A549 human alveolar epithelial carcinoma cells during *Mycoplasma pneumoniae* infection. BMC Microbiol. 14:27. doi: 10.1186/1471-2180-14-27, PMID: 24507763 PMC3922035

[ref21] Li X. Song Y. Wang X. Fu C. Zhao F. Zou L.. (2023). The regulation of cell homeostasis and antiviral innate immunity by autophagy during classical swine fever virus infection. Emerg. Microbes Infect. 12:2164217. doi: 10.1080/22221751.2022.2164217, PMID: 36583373 PMC9848339

[ref22] Liao Y. Peng K. Li X. Ye Y. Liu P. Zeng Y. (2022). The adhesion protein of *Mycoplasma genitalium* inhibits urethral epithelial cell apoptosis through CypA-CD147 activating PI3K/ Akt/NF-κB pathway. Appl. Microbiol. Biotechnol. 106, 6657–6669. doi: 10.1007/s00253-022-12146-z, PMID: 36066653

[ref23] Lin M. Liu H. Xiong Q. Niu H. Cheng Z. Yamamoto A.. (2016). Ehrlichia secretes Etf-1 to induce autophagy and capture nutrients for its growth through RAB5 and class III phosphatidylinositol 3-kinase. Autophagy 12, 2145–2166. doi: 10.1080/15548627.2016.1217369, PMID: 27541856 PMC5103349

[ref24] Liu Z. Wang T. Sun X. Nie M. (2023). Autophagy and apoptosis: regulatory factors of chondrocyte phenotype transition in osteoarthritis. Hum. Cell 36, 1326–1335. doi: 10.1007/s13577-023-00926-2, PMID: 37277675

[ref25] Ma Z. Zhang W. Wu Y. Zhang M. Wang L. Wang Y.. (2021). Cyclophilin a inhibits A549 cell oxidative stress and apoptosis by modulating the PI3K/Akt/mTOR signaling pathway. Biosci. Rep. 41:BSR20203219. doi: 10.1042/bsr20203219, PMID: 33393627 PMC7846964

[ref26] Mernaugh G. R. Dallo S. F. Holt S. C. Baseman J. B. (1993). Properties of adhering and nonadhering populations of *Mycoplasma genitalium*. Clin. Infect. Dis. 17, S69–S78. doi: 10.1093/clinids/17.supplement_1.s69, PMID: 8399942

[ref27] Miller D. R. Thorburn A. (2021). Autophagy and organelle homeostasis in cancer. Dev. Cell 56, 906–918. doi: 10.1016/j.devcel.2021.02.010, PMID: 33689692 PMC8026727

[ref28] Peng Y. Li C. Zhang L. Yu R. Wang Y. Pan L.. (2024). Cyclophilin a promotes porcine deltacoronavirus replication by regulating autophagy via the Ras/AKT/NF-κB pathway. Vet. Microbiol. 297:110190. doi: 10.1016/j.vetmic.2024.110190, PMID: 39084161

[ref29] Tamarelle J. Thiébaut A. C. M. de Barbeyrac B. Bébéar C. Ravel J. Delarocque-Astagneau E. (2019). The vaginal microbiota and its association with human papillomavirus, *Chlamydia trachomatis*, Neisseria gonorrhoeae and *Mycoplasma genitalium* infections: a systematic review and meta-analysis. Clin. Microbiol. Infect. 25, 35–47. doi: 10.1016/j.cmi.2018.04.019 29729331 PMC7362580

[ref30] Trent M. Coleman J. S. Hardick J. Perin J. Tabacco L. Huettner S.. (2018). Clinical and sexual risk correlates of *Mycoplasma genitalium* in urban pregnant and non-pregnant young women: cross-sectional outcomes using the baseline data from the Women’s BioHealth study. Sex. Transm. Infect. 94, 411–413. doi: 10.1136/sextrans-2017-053367, PMID: 29599387 PMC6170885

[ref31] Tully J. G. Taylor-Robinson D. Cole R. M. Rose D. L. (1981). A newly discovered mycoplasma in the human urogenital tract. Lancet 1, 1288–1291. doi: 10.1016/s0140-6736(81)92461-2, PMID: 6112607

[ref32] Ueno P. M. Timenetsky J. Centonze V. E. Wewer J. J. Cagle M. Stein M. A.. (2008). Interaction of *Mycoplasma genitalium* with host cells: evidence for nuclear localization. Microbiology 154, 3033–3041. doi: 10.1099/mic.0.2008/020735-0, PMID: 18832309

[ref33] Wang X. R. Cull B. (2022). Apoptosis and autophagy: current understanding in tick-pathogen interactions. Front. Cell. Infect. Microbiol. 12:784430. doi: 10.3389/fcimb.2022.784430, PMID: 35155277 PMC8829008

[ref34] Wang X. Fan L. Wang X. Luo T. Liu L. (2022). Cyclophilin a contributes to shikonin-induced glioma cell necroptosis and promotion of chromatinolysis. Sci. Rep. 12:14675. doi: 10.1038/s41598-022-19066-y, PMID: 36038617 PMC9424531

[ref35] Wang J. Hu K. Cai X. Yang B. He Q. Wang J.. (2022). Targeting PI3K/AKT signaling for treatment of idiopathic pulmonary fibrosis. Acta Pharm. Sin. B 12, 18–32. doi: 10.1016/j.apsb.2021.07.023, PMID: 35127370 PMC8799876

[ref36] Wang C. Liu Z. Y. Huang W. G. Yang Z. J. Lan Q. Y. Fang A. P.. (2024). Choline suppresses hepatocellular carcinoma progression by attenuating AMPK/mTOR-mediated autophagy via choline transporter SLC5A7 activation. Hepatobil. Surg. Nutr. 13, 393–411. doi: 10.21037/hbsn-22-476, PMID: 38911213 PMC11190510

[ref37] Wu Y. Ma Z. Zhang Y. Zhang M. Zhang W. Zhang M.. (2022). Cyclophilin a regulates the apoptosis of A549 cells by stabilizing Twist1 protein. J. Cell Sci. 135:jcs259018. doi: 10.1242/jcs.259018, PMID: 34881782

[ref38] Xu Z. Han X. Ou D. Liu T. Li Z. Jiang G.. (2020). Targeting PI3K/AKT/mTOR-mediated autophagy for tumor therapy. Appl. Microbiol. Biotechnol. 104, 575–587. doi: 10.1007/s00253-019-10257-8, PMID: 31832711

