# Supplementary figures and images for: Mycoplasma genitalium protein of adhesion inhibits human urethral epithelial cells apoptosis via CypA/PI3K/AKT/mTOR-dependent autophagy
Source: Front Microbiol. 2025 Mar 26;16:1570659. doi: 10.3389/fmicb.2025.1570659 (PMC11979137; doi:10.3389/fmicb.2025.1570659)

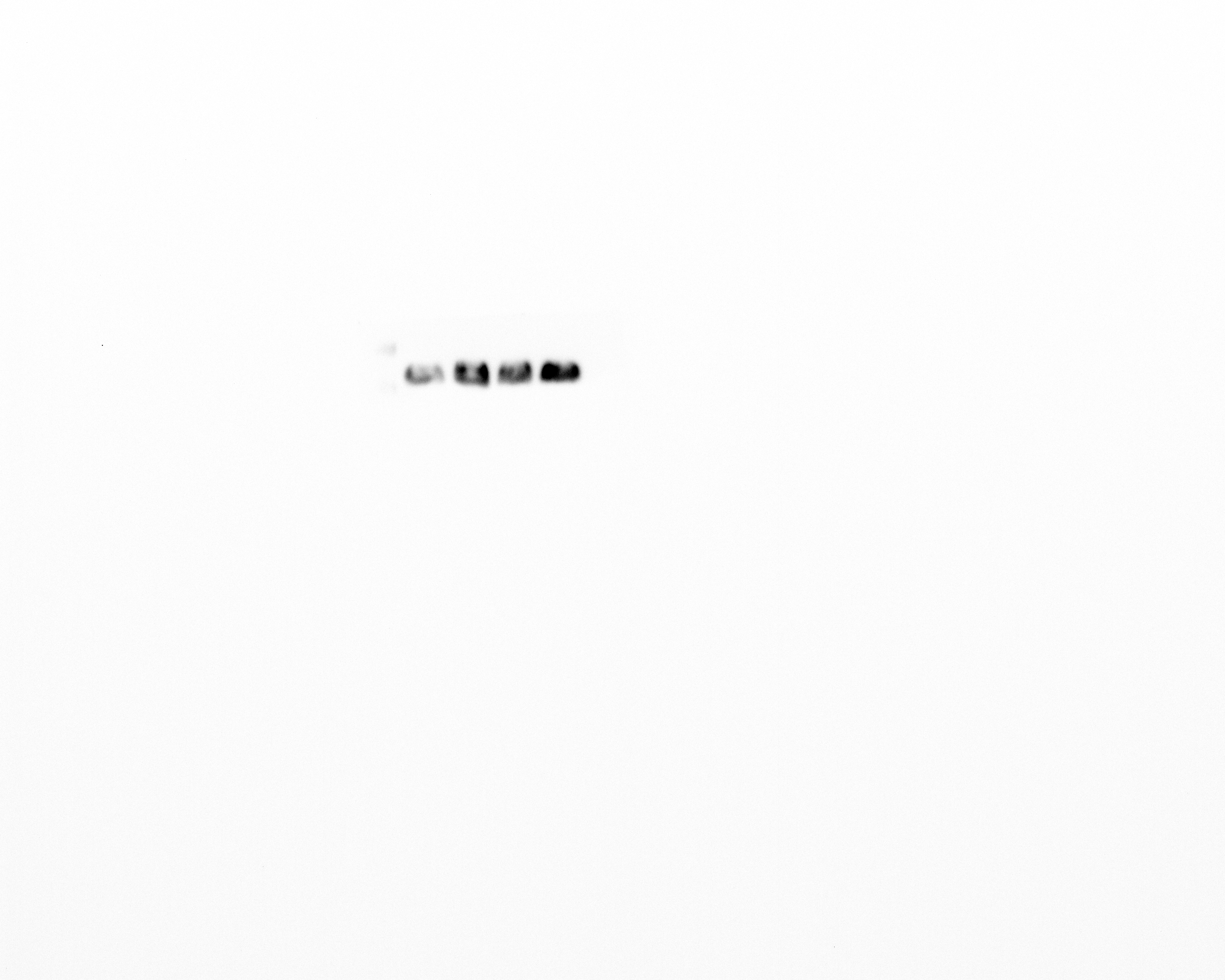

Supplement: Supplementary file 1 [file Presentation_1.zip › SFig.1 - 副本/1. Sig.1—CypA.tif]

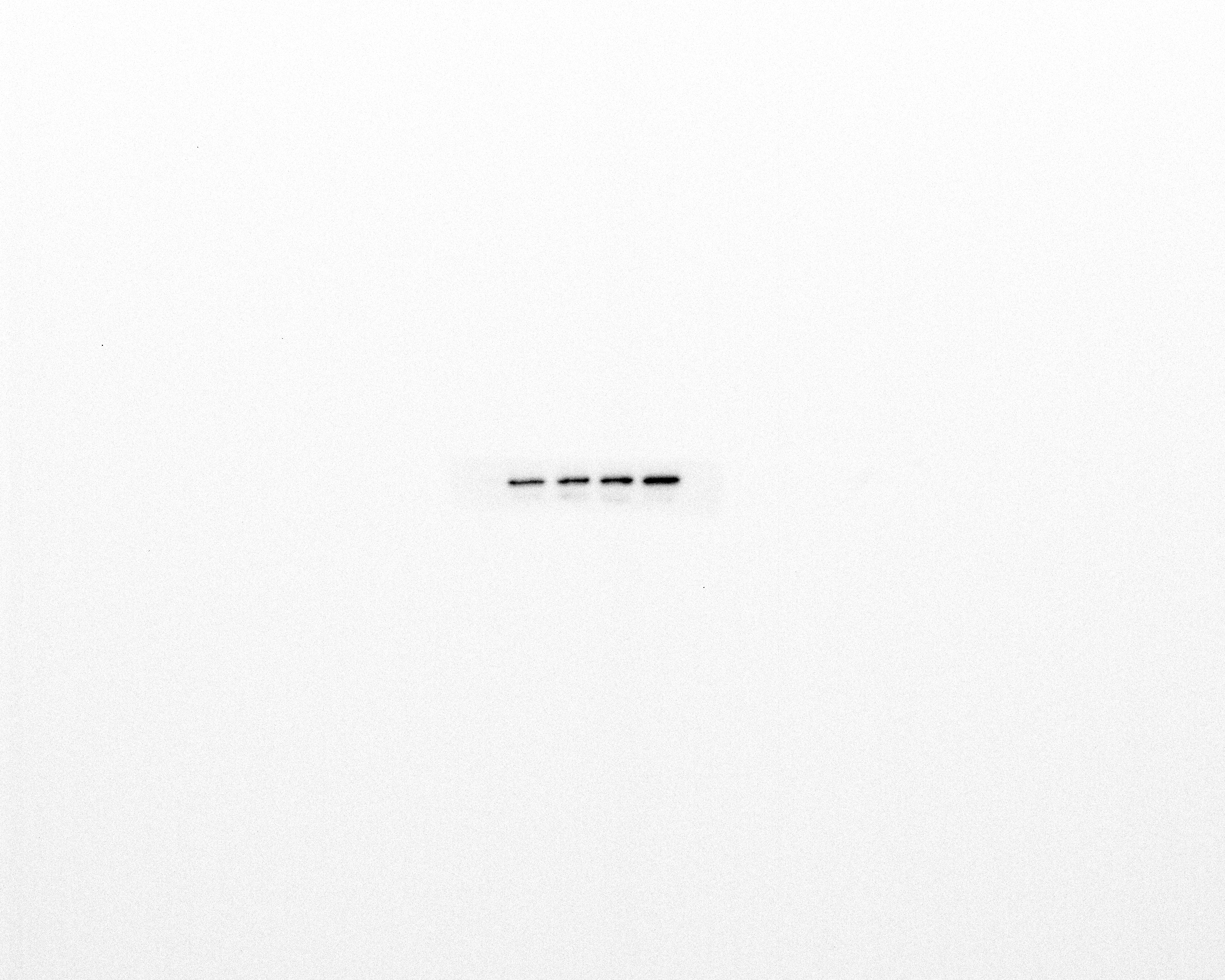

Supplement: Supplementary file 1 [file Presentation_1.zip › SFig.1 - 副本/2. SFig.1—β-actin.tif]

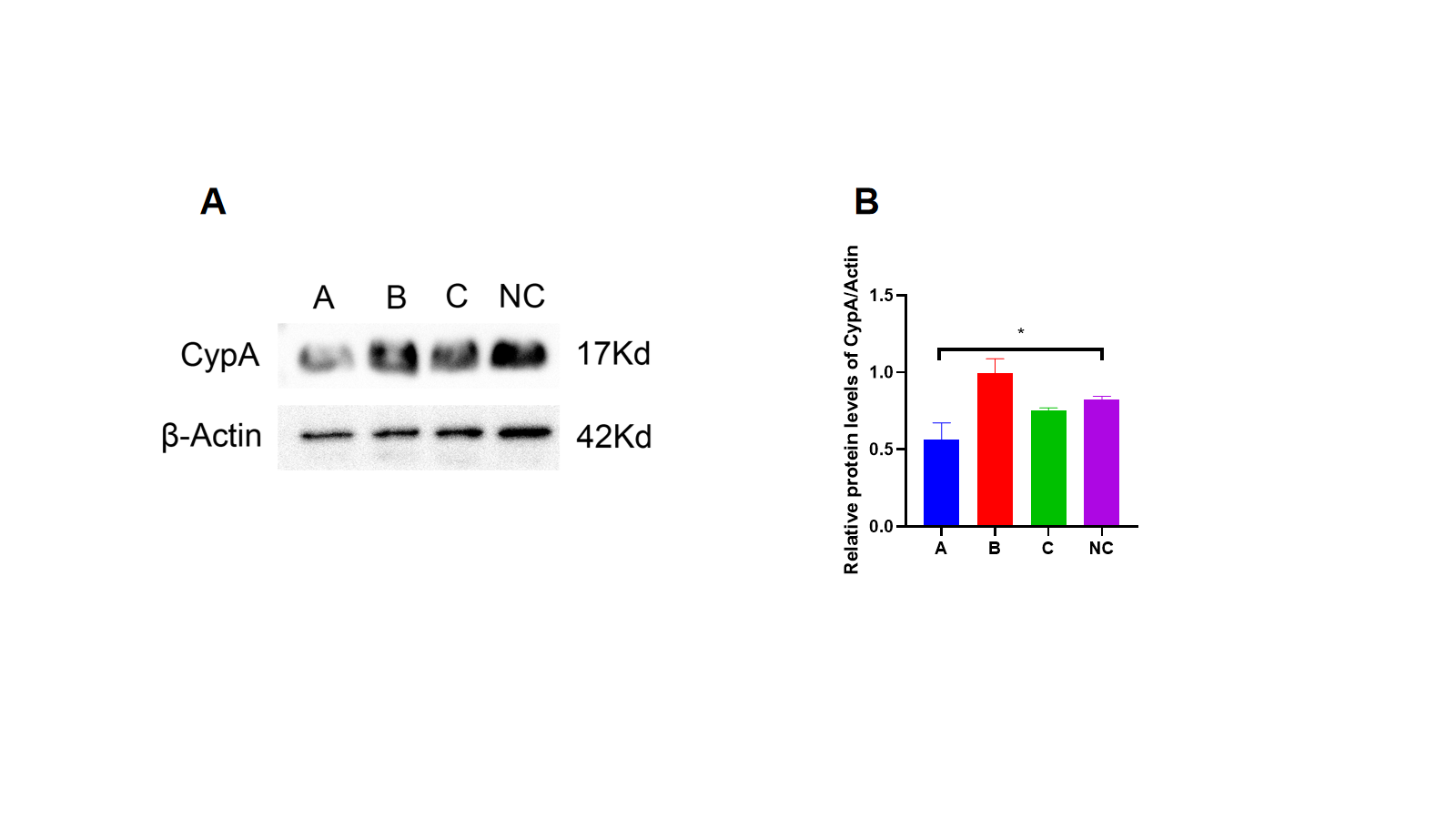

Supplement: Supplementary file 1 [file Presentation_1.zip › SFig.1 - 副本/3. SFig.1-analysis.tif]
